# Cholesterol Homeostasis in Two Commonly Used Human Prostate Cancer Cell-Lines, LNCaP and PC-3

**DOI:** 10.1371/journal.pone.0008496

**Published:** 2009-12-30

**Authors:** James Robert Krycer, Ika Kristiana, Andrew John Brown

**Affiliations:** School of Biotechnology and Biomolecular Sciences, University of New South Wales, Sydney, New South Wales, Australia; New Mexico State University, United States of America

## Abstract

**Background:**

Recently, there has been renewed interest in the link between cholesterol and prostate cancer. It has been previously reported that *in vitro*, prostate cancer cells lack sterol-mediated feedback regulation of the major transcription factor in cholesterol homeostasis, sterol-regulatory element binding protein 2 (SREBP-2). This could explain the accumulation of cholesterol observed in clinical prostate cancers. Consequently, perturbed feedback regulation to increased sterol levels has become a pervasive concept in the prostate cancer setting. Here, we aimed to explore this in greater depth.

**Methodology/Principal Findings:**

After altering the cellular cholesterol status in LNCaP and PC-3 prostate cancer cells, we examined SREBP-2 processing, downstream effects on promoter activity and expression of SREBP-2 target genes, and functional activity (low-density lipoprotein uptake, cholesterol synthesis). In doing so, we observed that LNCaP and PC-3 cells were sensitive to increased sterol levels. In contrast, lowering cholesterol levels via statin treatment generated a greater response in LNCaP cells than PC-3 cells. This highlighted an important difference between these cell-lines: basal SREBP-2 activity appeared to be higher in PC-3 cells, reducing sensitivity to decreased cholesterol levels.

**Conclusion/Significance:**

Thus, prostate cancer cells are sensitive to changing sterol levels *in vitro*, but the extent of this regulation differs between prostate cancer cell-lines. These results shed new light on the regulation of cholesterol metabolism in two commonly used prostate cancer cell-lines, and emphasize the importance of establishing whether or not cholesterol homeostasis is perturbed in prostate cancer *in vivo*.

## Introduction

The study of cholesterol homeostasis in a prostate cancer (PCa) setting began in 1942, when Swyer published *in situ* findings of elevated cholesterol levels in benign prostatic hyperplasia compared to normal tissue [1]. More recently, there has been renewed interest in the links between cholesterol and PCa [2]–[4]. For instance, it has been proposed that an in-depth understanding of cholesterol regulation in PCa progression may lead to the development of novel drug targets [5]. In line with this, several epidemiological studies have reported an association between the use of statins (cholesterol-lowering drugs) and reduced risk of advanced PCa (reviewed in [2]–[4]).

Cholesterol has an important influence on membrane integrity, signaling, and metabolism, and thus there is a need to regulate its levels within the cell [2]. One major homeostatic mechanism occurs at the transcriptional level, via the master transcription factor: sterol-regulatory element binding protein 2 (SREBP-2). The regulation of this transcription factor has been reviewed by Brown and Goldstein [6]. Briefly, SREBP-2 is synthesized as a precursor, bound to the endoplasmic reticulum (ER). When cholesterol levels are low, SREBP-2 is transported from the ER to the Golgi apparatus, where it is processed to release the N-terminal domain. This mature form of SREBP-2 migrates into the nucleus, where it upregulates cholesterogenic genes, such as those encoding the low-density lipoprotein receptor (LDLR) and 3-hydroxy-3-methylglutaryl coenzyme A reductase (HMGCR). This promotes the uptake and synthesis of cholesterol until cholesterol levels are sufficient, after which SREBP-2 is retained in the ER, preventing its activation and thus downregulating target gene expression. This sterol-dependent feedback mechanism also regulates the SREBP-1a/c isoforms. In general, SREBP-1c preferentially upregulates fatty-acid-related genes, SREBP-2 targets cholesterol-related genes, and SREBP-1a can activate both [7], [8].

It has been suggested that that this feedback regulation of SREBP-2 is lacking in PCa, through the observation that treatment with sterols reduced SREBP-2 target gene expression, as well as low-density lipoprotein (LDL) uptake, in normal cells but not in PC-3 and DU145 PCa cells *in vitro* [9]. Perturbations in sterol-mediated feedback would explain the accumulation of cholesterol in PCa specimens [10], [11], and has become a widely-accepted concept in the PCa setting (reviewed in [3], [4]). This dysregulation implies that PCa cells, and perhaps cancer cells in general (for example, [9], [12]–[15]), are a special case in that they do not conform to the currently-held paradigm of cellular cholesterol homeostasis [16]. An accumulation of cholesterol within the cell would, for instance, stiffen the mitochondrial membrane, reducing oxidative phosphorylation – this promotes glycolysis even in the presence of oxygen (the Warburg effect [17]), a metabolic phenotype commonly observed in cancer cells and of great interest in cancer research [18].

The aim of our investigation was to explore cholesterol regulation in greater depth in two commonly used PCa cell-lines, PC-3 and LNCaP, using a variety of conditions and approaches. We sought to confirm previous findings of SREBP-2 activity being unaffected by sterols [9], and determine if this dysregulation affects the response of PCa cells to lowered sterol levels. From our results, we provide a new perspective on cholesterol homeostasis in these PCa cell-lines, having implications for both laboratory experiments and PCa therapy.

## Results

### Sterol-mediated regulation of SREBP-2 target genes exists in prostate cancer cells

To examine sterol regulation of SREBP-2 in the first instance, we analyzed the mRNA expression of two SREBP-2 target genes (*LDLR*, *HMGCR*) upon manipulating the cholesterol status of PC-3 and LNCaP cells. Experiments were conducted under lipoprotein-deficient conditions (with lipoprotein-deficient fetal calf serum [FCLPDS]) to enhance the effects of treating cells with 25-hydroxycholesterol (25-HC), an oxygenated cholesterol derivative (oxysterol), and LDL. LDL delivers cholesterol via the LDLR, presenting a physiological alternative for increasing intracellular sterol levels.

**Figure 1:**
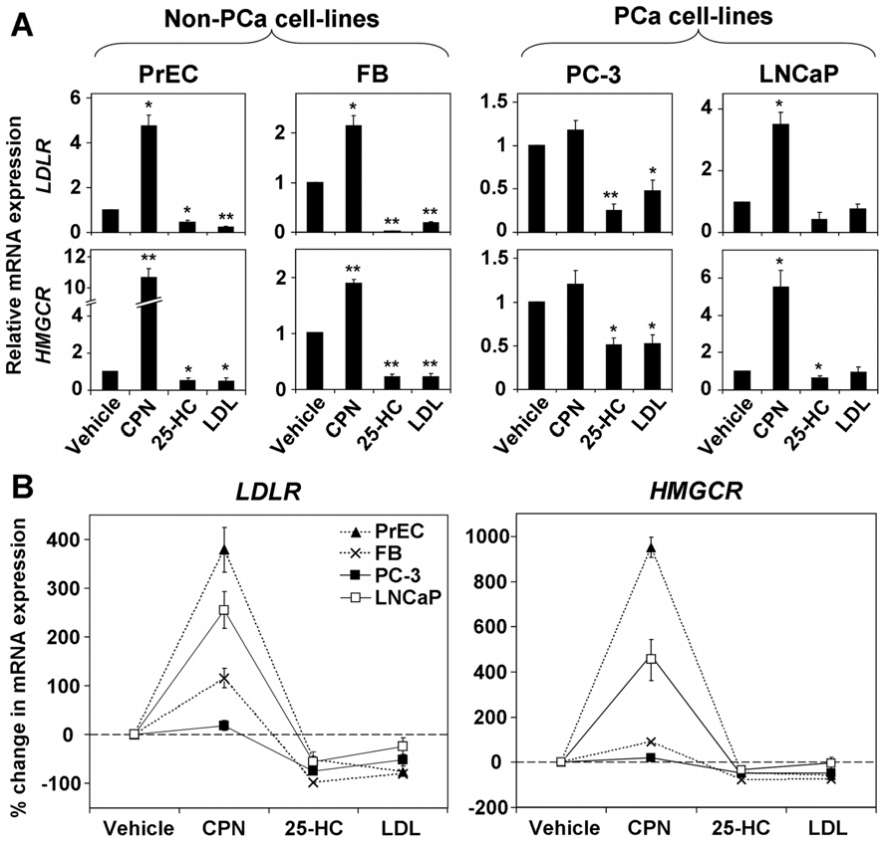
Sterol-mediated regulation of SREBP-2 target genes exists in PCa cells. Cells were treated with the statin compactin (CPN, 5 µM), oxysterol 25-HC (1 µg/ml), or LDL (50 µg/ml) for 24 h. Cellular RNA was harvested and mRNA expression of the *LDLR* and *HMGCR* genes was analyzed by qRT-PCR. The mRNA levels were made relative to the vehicle condition as described in Materials and Methods. Data are mean + SEM, from 3 separate experiments for each cell-line. Each experiment was performed with triplicate wells per condition. (A) Data presented separately for each cell-line. * *p*<0.05, ** *p*<0.01, two-sample *t*-test versus vehicle condition. (B) Data from (A) has been overlaid for each gene, represented as mean±SEM for each datapoint. The PCa cell-lines are represented by solid lines, whilst the non-PCa cell-lines are represented by broken lines.

If regulation of SREBP-2 is present, the addition of sterols, through either 25-HC or LDL treatment, would reduce SREBP-2 processing and SREBP-2 target gene expression. On the other hand, the statin compactin (also known as mevastatin) inhibits HMGCR, which catalyses a rate-limiting step in cholesterol synthesis, and thus should increase SREBP-2 activity. The non-cancerous cell-lines, prostate epithelial cells (PrEC) and fibroblasts (FB), served as a control, demonstrating both forms of feedback regulation (Fig. 1A). Similar sterol-mediated regulation was also observed in the PCa cell-lines (Fig. 1A), contrary to previous findings [9].

In PC-3 cells, increasing cellular sterol status with 25-HC or LDL significantly reduced lipogenic gene expression, in comparison to the vehicle condition (Fig. 1A). The similarity between vehicle- and compactin-treated PC-3 cells is unlikely due to resistance to compactin, since we found that compactin inhibits cholesterol synthesis in PC-3 cells by metabolic labeling (data not shown). In contrast, compactin significantly increased lipogenic gene expression in LNCaP cells and sterol-treated LNCaP cells had similar expression patterns to the vehicle-treated cells (Fig. 1A). Overlaying the relative mRNA expression data from each cell-line (Fig. 1B) showed that the PCa cells (solid lines) had a reduced response to compactin compared to PrEC cells, and were less affected by LDL than both non-PCa cell-lines. Nevertheless, these PCa cell-lines were both sensitive to changes in cholesterol status.

### Sterol-mediated regulation is specific to SREBP-2

Given that the expression of two SREBP-2 target genes (*LDLR*, *HMGCR*) responded similarly to changing cholesterol status (Fig. 1), we sought more direct evidence that this effect was mediated by the SREBP-2 transcription factor.

**Figure 2:**
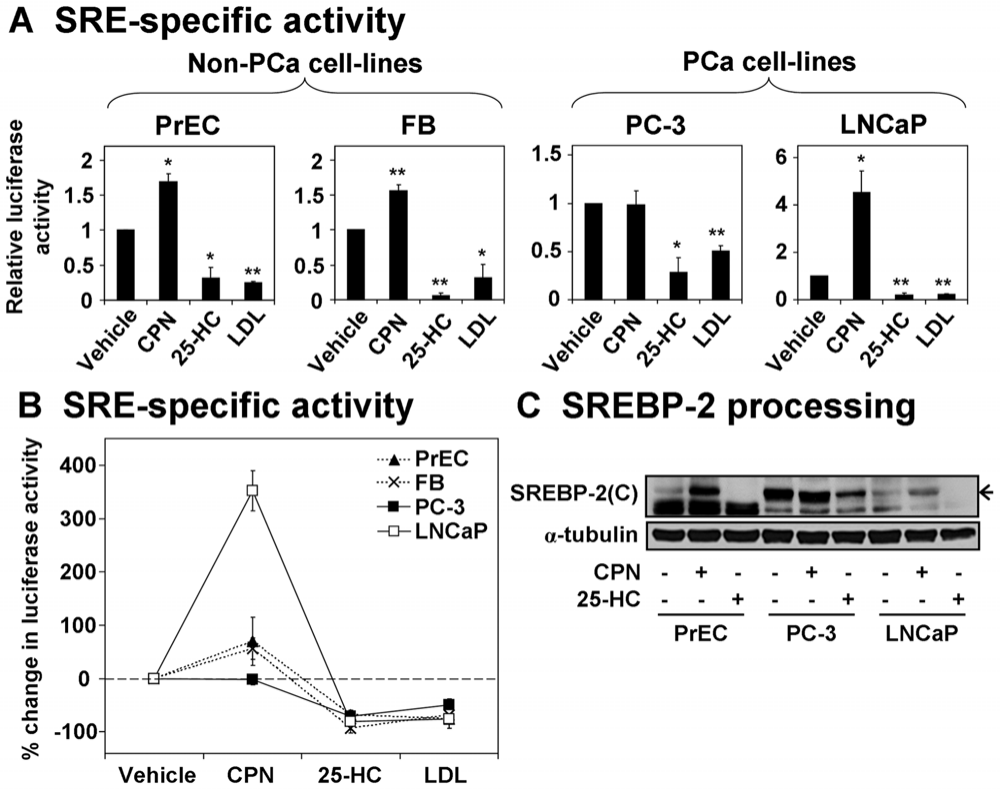
Responses to changing sterol status involve SREBP-2 activation in prostate cancer cells. (A) Cells were transfected as described in Materials and Methods. Treatment included the statin compactin (CPN, 5 µM), oxysterol 25-HC (1 µg/ml), or LDL (50 µg/ml) for 24 h. SRE-specific luciferase activity was determined as described in Materials and Methods, and normalized to the vehicle condition. The wildtype and mutant promoter values are shown in Fig. S1. Data are mean + SEM, from 3 separate experiments for each cell-line. Each experiment was performed with triplicate wells per condition. * *p*<0.05, ** *p*<0.01, two-sample *t*-test versus vehicle condition. (B) Data from (A) has been overlaid, represented as mean±SEM for each datapoint. The PCa cell-lines are represented by solid lines, whilst the non-PCa cell-lines are represented by broken lines. (C) Cells were treated with CPN (5 µM) or 25-HC (1 µg/ml) for 24 h. Cell lysates were subjected to SDS-PAGE and Western blotted with the IgG-1C6 anti-SREBP-2 antibody. The C-terminal cleavage product of SREBP-2, SREBP-2(C), is labeled with an arrow – we assume that the band below is a non-specific band. Probing for α-tubulin served as an internal loading control. The blot shown is representative of at least 2 separate experiments for each cell-line.

Since mature SREBP-2 binds to the sterol-regulatory element (SRE) within a target gene's promoter region, we developed an SRE-specific luciferase assay, utilizing the LDLp-588luc plasmid [19], which encodes firefly luciferase under the transcriptional regulation of the *LDLR* promoter. Using site-directed mutagenesis, we disrupted the SRE within the promoter region to generate a negative control plasmid, LDLp-mutSRE. We found that the wild-type promoter (LDLp-588luc) exhibited the predicted changes in luciferase activity (increasing with compactin treatment, decreasing with 25-HC or LDL treatment), whilst the mutant promoter (LDLp-mutSRE) produced negligible changes (Fig. S1). The mutant promoter luciferase activity was subtracted from that of the wild-type promoter for each treatment condition to obtain SRE-specific activity. This luciferase assay revealed that feedback regulation occurred in PCa cells in response to altered cholesterol levels (Fig. 2A).

In LNCaP cells, SRE-specific activity (Fig. 2A) appeared more sensitive than SREBP-2 target gene expression (Fig. 1) to sterol treatment. Consequently, overlaying the data for each cell-line (Fig. 2B) revealed that each cell-line was affected similarly by sterol treatment. In contrast, compactin treatment again demonstrated that PC-3 and LNCaP cells differ in the extent of their homeostatic responses: SRE-specific activity was greatly increased in LNCaP cells compared to the non-PCa cell-lines (dotted lines), in contrast to PC-3 cells (Fig. 2B). Interestingly, in PrEC cells, the response to compactin (Fig. 2A) was blunted in comparison to SREBP-2 target gene expression (Fig. 1A). This suggests that other transcription factors may alter the effects of SREBP-2 on target gene expression, justifying the use of the SRE-specific luciferase assay.

However, the SREBP-1a isoform also binds to the same SREs as SREBP-2 with strong affinity [7], potentially confounding these results. Hence, we tested whether this effect was SREBP-2-specific by Western blotting. Since the IgG-1C6 anti-SREBP-2 antibody binds to the C-terminus [20], it detects the C-terminal cleavage product, giving an indication of SREBP-2 processing. For instance, sterols would promote the retention of SREBP-2 precursor in the ER [21], reducing cleaved SREBP-2. We found that 25-HC reduced SREBP-2 cleavage in all three prostate cell-lines, whilst compactin increased SREBP-2 cleavage in PrEC and LNCaP cells, but not in PC-3 cells (Fig. 2C). Thus, the degree of SREBP-2 processing correlated with the regulation of promoter activity (Fig. 2A,B) and SREBP-2 target gene expression (Fig. 1) observed in these PCa cell-lines. Overall, this data shows that PC-3 and LNCaP cells are both sensitive to sterols, but differ in their responses to compactin treatment.

### Changes in SREBP-2 activity translate to the functional level in PCa cells

To see if transcriptional regulation exerts homeostatic effects on cholesterol metabolism in PCa cells, we examined the effects of altering sterol status on LDLR activity and cholesterol synthesis.

**Figure 3:**
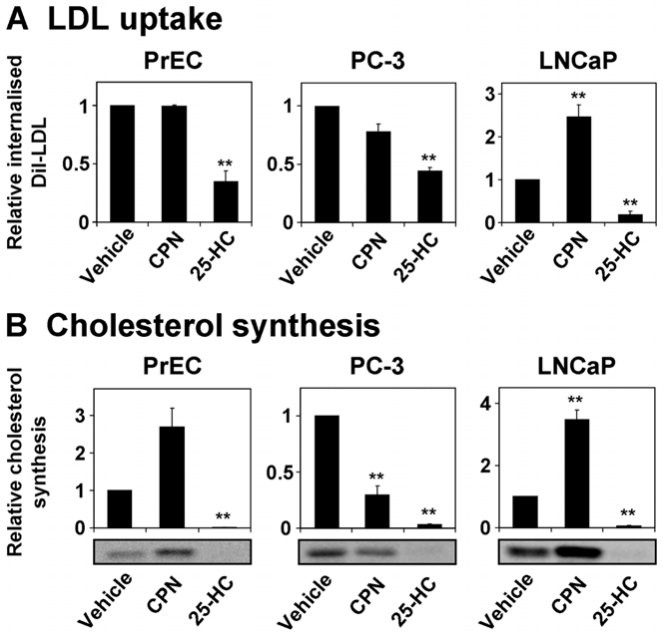
Sterol feedback regulation has functional effects in PCa cells. Cells were treated with the statin compactin (CPN, 5 µM) or the oxysterol 25-HC (1 µg/ml) for 24 h in Medium C. (A) Cells were prepared, treated, and assayed for DiI-LDL internalization as described in Materials and Methods. The amount of DiI-LDL internalized provides an indication of LDLR activity. Data are presented as mean + SEM, from at least 3 separate experiments for each cell-line. Each experiment was performed with triplicate wells per condition. (B) After treatment, cells were washed with PBS and radiolabeled with [1-^14^C]-acetic acid for 2 h. Radiolabeling was performed in the presence of treatment, with the exception of the CPN treatment (in which case the CPN was absent). Cells were then harvested and lipid extracts were subjected to thin layer chromatography and phosphorimaging as described in Materials and Methods. The phosphorimages shown are representative of at least 3 separate experiments for each cell-line. Densitometry was performed and data presented as mean+SEM for each cell-line. * *p*<0.05, ** *p*<0.01, two-sample *t*-test versus vehicle condition.

The activity of LDLR was determined using an LDL uptake assay. Following incubation with DiI-LDL (LDL labeled with the fluorescent dye DiI), the subsequent fluorescence of the cells provided an indication of LDL uptake. Since LDL is internalized at 37°C, but not 4°C [22], each experiment was performed twice simultaneously, with one set of cells incubated with DiI-LDL at 37°C and the other at 4°C – the difference in fluorescence between the two sets provided a measure of internalized DiI-LDL. This also controlled for non-specific binding of DiI-LDL. This assay revealed that, relative to the vehicle condition, 25-HC caused a significant decrease in DiI-LDL internalization in all cell-types (Fig. 3A). In contrast, compactin increased LDLR activity in LNCaP cells only, having no significant effect in PrEC cells and causing a decrease (albeit not significant, *p* = 0.08) in PC-3 cells (Fig. 3A).

To determine cholesterol synthesis, cells were radiolabeled after treatment. The 25-HC treatment was maintained during radiolabeling, whilst the compactin treatment was removed – since compactin would reduce cholesterol synthesis, we instead attempted to simulate the ‘statin rebound effect’ [23]. This phenomenon is a homeostatic response to compactin: normally, compactin increases SREBP-2 processing (Fig. 2B), upregulating the expression of cholesterol synthetic enzymes. Consequently, when compactin is removed, due to the high levels of enzymes present, there will be a large flux through the pathway. This ironically causes an acute increase in cholesterol levels. This can be observed in PrEC and LNCaP cells (Fig. 3B). However, compactin pre-treatment surprisingly reduced cholesterol synthesis during radiolabeling in PC-3 cells (Fig. 3B). Despite this, 25-HC abolished cholesterol synthesis in all three cell-lines (Fig. 3B).

Taken together, these data demonstrate that regulation of both cholesterol uptake (via LDL) and synthesis are sensitive to sterol levels in PCa cells.

### Basal SREBP-2 activity is higher in PC-3 cells than in LNCaP cells

**Figure 4:**
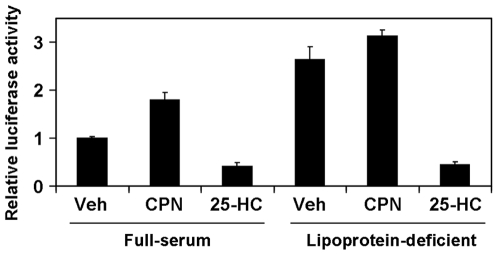
SRE-specific activity is saturated under lipoprotein-deficient conditions in PC-3 cells. PC-3 cells were transfected as described in Materials and Methods. Treatment included the statin compactin (CPN, 5 µM) or oxysterol 25-HC (1 µg/ml) for 24 h, in either full-serum (Medium A) or lipoprotein-deficient serum (Medium C). SRE-specific luciferase activity was determined as described in Materials and Methods, and normalized to the vehicle condition. Data are mean + SD, representative of 2 separate experiments. Each experiment was performed with triplicate wells per condition.

Whilst both PC-3 and LNCaP cell-lines are sterol-responsive, SREBP-2 activity in PC-3 cells appeared less sensitive to lowered cholesterol levels (compactin treatment, Figs. 1, 2). These experiments were conducted under lipoprotein-deficient conditions (Medium C), whilst a two-fold increase in SRE-specific activity was observed with compactin treatment (relative to the vehicle treatment) under full-serum conditions (Fig. 4). This suggests that in PC-3 cells, the compactin effect is masked by ‘saturation’ of SREBP-2 processing in lipoprotein-deficient media, such that further cholesterol deprivation via compactin treatment would have little effect. This further implies that PC-3 cells are not insensitive to compactin *per se*, but require less sterol deprivation to maximize SREBP-2 activity compared to other cell-lines, including the LNCaP cells.

**Figure 5:**
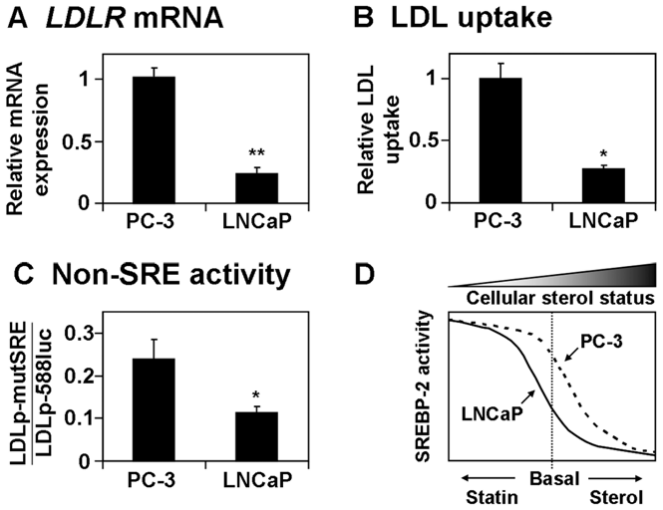
Basal LDLR gene expression and activity is higher in PC-3 than LNCaP cells. Data was pooled from experiments where LNCaP and PC-3 cells received vehicle treatment in Medium C for 24 h. (A) For each qRT-PCR experiment, the ΔC_t_ values (for threshold = 10^−1.5^ normalized fluorescence units) for the *LDLR* gene, relative to the *PBGD* housekeeping gene, were considered. Expression is represented as a fold change (relative to PC-3 cells), whereby a one-unit increase in ΔC_t_ results in a two-fold decrease in mRNA expression. Data are presented as mean + SEM, from at least 4 separate experiments for each cell-line. (B) Raw LDL uptake, measured as fluorescence normalised by protein content in the LDL uptake assay, was averaged between experiments and made relative to the PC-3 cells. Data are presented as mean + SEM, from at least 3 separate experiments for each cell-line. (C) For each SRE-specific luciferase assay, the firefly/*Renilla* luciferase ratios generated from the LDLp-mutSRE plasmid were normalised to that of the LDLp-588luc plasmid. Data presented as mean+SEM, from at least 5 separate experiments for each cell-line. Each experiment in (A)–(C) was performed with triplicate wells per condition. * *p*<0.05, ** *p*<0.01, two-sample *t*-test versus PC-3 cells. (D) A model depicting the differences in cholesterol homeostasis between PC-3 and LNCaP cells. The threshold level of cellular cholesterol, at which SREBP-2 activity becomes dramatically reduced, may be higher in PC-3 cells. This would reduce the effect of statins, relative to the basal condition.

This important difference between LNCaP and PC-3 cells was explored further. As a case-study, the baseline regulation and activity of LDLR were considered under lipoprotein-deficient conditions, from experiments described in Figs 1–3. Firstly, mRNA expression data was considered: the ΔC_t_ values for a threshold of 10^−1.5^ normalized fluorescence units were compared between PC-3 and LNCaP cells. The C_t_ values for the housekeeping gene, porphobilinogen deaminase (*PBGD*), were similar between the two cell-lines (*p*≈0.73), justifying this comparison. The average *LDLR* ΔC_t_ value was ∼2 units lower in PC-3 cells, indicating a 4-fold higher basal *LDLR* mRNA expression than LNCaP cells (Fig. 5A). Subsequently, basal LDLR activity was also significantly higher in PC-3 cells (Fig. 5B).

Together with Fig. 2C, this implies that basal SREBP-2 activity is higher in PC-3 cells. However, other transcription factors may also contribute to the expression of SREBP-2 target genes. Hence, previous SRE-specific luciferase experiments were re-analysed. For the vehicle condition, the relative luciferase activity generated by the LDLp-mutSRE (mutated SRE in *LDLR* promoter) was considered as a proportion of that of LDLp-588luc (wildtype *LDLR* promoter). The mutSRE-fluc luciferase activity was assumed to represent non-SRE activity since compactin and 25-HC treatment had little effect on LDLp-mutSRE activity in all cell-lines (Fig. S1). The mutSRE-fluc/LDLR-fluc proportion was higher in PC-3 cells than LNCaP cells (Fig. 5C), implying other transcription factors may also contribute to the differences in *LDLR* mRNA expression observed between these cell-lines (Fig. 5A). Taken together, these data suggest that basal activity downstream of SREBP-2 is upregulated in PC-3 cells, resulting in a maximal response under basal conditions.

## Discussion

In this investigation, we sought to gain insight into cellular cholesterol homeostasis in the PCa setting. An aberrant feedback-response to sterols appears to be a common phenomenon in cancer cells [9], [12]–[15] and would explain the accumulation of cholesterol in clinical PCa [10], [11]. Our findings suggest that sterol-regulated processing of SREBP-2 exists in PCa cells (Fig. 2C). Consequently, sterol feedback regulation had downstream effects at the SRE (Fig. 2B), on the expression of SREBP-2 target genes (Fig. 1B), and at the functional level (Fig. 3). We also considered the response of these cells to reduced sterol levels, finding that PC-3 and LNCaP cells differed in their degrees of regulation (Fig. 5).

In PC-3 cells, sterols caused a significant decrease in SREBP-2 activity, conflicting with previous findings [9]. Besides methodological considerations, such as quantitative versus semi-quantitative methods of mRNA determination, other factors may account for the discrepancy with their findings. For instance, it has been shown that colonic adenocarcinoma cells were unaffected by sterols at higher densities, but demonstrated feedback regulation when plated at lower densities [14]. Cells at lower densities are exponentially growing, with higher cholesterol uptake and synthesis rates found in both cancerous and normal cells [14], [24]. This could allow for the reconciliation of the findings here with those of the previous study [9], particularly since their experiments were run for a longer duration (30 and 45 h, versus 24 h here). We plated PC-3 cells at low density, to prevent overconfluence at 48 h, and found sensitivity to sterols up to 48 h (Fig. S2).

Similarly, sterols were found to reduce SREBP-2 activity in LNCaP cells (Fig. 2). Here, there was a slight decrease in SREBP-2 target gene expression (Fig. 1), whilst cholesterol uptake and synthesis was abolished (Fig. 3). This is supported by a previous finding that sterols downregulated the expression of HMG-CoA synthase, another SREBP-2 target, in LNCaP cells [25]. Hence, PCa cells are indeed sensitive to increased sterol levels. This does not negate the idea of disrupted sterol-feedback in PCa cells, but rather raises more questions: do laboratory PCa cell-lines accumulate cholesterol as is seen in clinical PCa [10], [11]? Would PCa cells be less sensitive to sterols in an *in vivo* context, such as in xenografts? Clearly, this warrants further investigation.

We also examined the reverse situation, reducing cholesterol levels using the statin compactin. Similarly to PrEC cells, this increased SREBP-2 activity (Fig. 2), enhanced SREBP-2 target gene expression (Fig. 1), and induced a statin-rebound effect in cholesterol synthesis (Fig. 3B) in LNCaP cells. Interestingly, compactin did not affect LDLR activity in PrEC cells (Fig. 3A), whilst it has been proposed that statins reduce blood-cholesterol levels by increasing LDLR expression (seen here in Fig. 1A) and thus LDL uptake [26]. This apparent paradox may be explained since PCSK9 (proprotein convertase subtilisin/kexin type 9), another SREBP-2 target, has been found to promote LDLR degradation, limiting the effectiveness of statins [27], [28]. LNCaP cells appear to bypass this regulatory mechanism (Fig. 3A), demonstrating increased LDLR activity upon compactin treatment. Overall, this shows that LNCaP cells respond to low sterol levels.

In contrast, compactin did not cause a significant increase in SREBP-2 target gene expression (Fig. 1) in PC-3 cells, relative to the vehicle condition. Compactin was confirmed to inhibit cholesterol synthesis by metabolic labeling. Hence, the lack of compactin effect is likely due to near-maximal SREBP-2 processing from incubation in the lipoprotein-deficient media (Fig. 4). Supporting this, the basal levels of processed SREBP-2 appear to be highest in PC-3 cells (Fig. 2C), and basal LDLR expression and activity were higher in PC-3 than LNCaP cells (Fig. 5A,B). Thus, PC-3 cells require less sterol deprivation in order to invoke a maximum response from the SREBP-2 pathway. Furthermore, compactin treatment tended to reduce LDLR activity (Fig. 3A) and pretreatment lowered cholesterol synthesis (Fig. 3B), for reasons that are currently unclear.

Therefore, PC-3 and LNCaP cells vary in their cholesterol homeostasis. Radhakrishnan *et al.* [29] propose a ‘switch-like control’ of SREBP-2 activity, whereby a sharp drop in SREBP-2 processing occurs when intracellular (ER-)cholesterol levels reach a precise threshold. We propose that this ‘regulatory gauge’ is higher in PC-3 cells (Fig. 4D), accounting for 1) PC-3 cells having higher basal SREBP-2 than LNCaP cells, 2) statins appearing to have little effect in PC-3 cells (relative to the basal condition), and 3) sterols reducing SREBP-2 activity in both PCa cell-lines.

This difference in cholesterol regulation may be attributed to other phenotypic differences between these cell-lines, such as androgen responsiveness. LNCaP cells are androgen-sensitive [30], whilst PC-3 cells are relatively androgen-independent [31]. Androgens have been shown to upregulate SCAP expression, subsequently increasing SREBP-2 activation [32]. Since a lack of feedback regulation was previously observed in the androgen-independent PCa cell-lines (PC-3, DU145) [9], it has been argued that disrupted sterol feedback may be associated with androgen deprivation because SREBP-2 was upregulated in LNCaP xenografts *in vivo* upon host castration [33]. However, this cannot be reconciled with our results since PC-3 cells were found to be sterol-sensitive here. Nevertheless, a factor involved in androgen-independence may favor PC-3 cells, potentially raising baseline SREBP-2 activity. Alternatively, other transcription factors may contribute to SREBP-2 target gene expression (Fig. 5C), such as oncostatin M binding to the SIRE (sterol-independent response element) within the *LDLR* promoter [34], augmenting basal cholesterol metabolism in PC-3 cells. Further investigations are needed to delineate the precise mechanisms by which cholesterol homeostasis differs in these PCa cell-lines – in light of this, a recent paper has reported differences in the transcriptional profile between LNCaP and PC-3 cells [35], albeit not directly related to cholesterol homeostasis.

Consequently, our findings support the assertion that these two cell-lines cannot be treated as synonymous examples of PCa cell-lines for *in vitro* studies [35], [36]. However, it is difficult to relate these findings to a clinical setting because, to the best of our knowledge, no studies have examined the sterol-mediated regulation of SREBP-2 and cholesterol metabolism in cells isolated from prostate specimens. In addition, there have been conflicting reports on the expression profile of PCa in a sterol-related context. For instance, in recent studies examining the changing profile with progression to the metastatic, hormone-refractory state: some studies found an increase in the expression of SREBP-2 [37] and sterol biosynthetic [38] genes, whilst another found a decrease [39]. Such conflicts may result from differences in patient populations, sample preparations, or microarray platforms (reviewed in [40]).

Nevertheless, it has been argued that LNCaP cells are more characteristic of clinical PCa than PC-3 cells [41], [42]. Hence, the findings here may have clinical ramifications: in particular, LNCaP cells have higher LDLR activity in response to compactin treatment. This suggests that treatment with a cholesterol-lowering drug (such as a statin) may induce LDL uptake specifically in PCa cells. This implies that concurrent administration of such tumor-specific chemotherapeutic agents, incorporated into LDL or other LDLR-binding vesicles [43], [44], may provide a potential treatment option for PCa.

## Materials and Methods

### Materials

Non-cancerous human PrEC cells were obtained from Lonza (Mt Waverley, Vic, AU), human foreskin FB cells were a gift from Dr Ingrid Gelissen (University of New South Wales, AU), PC-3 cells were a gift from Dr Qihan Dong (University of Sydney, AU), and LNCaP cells were a gift from Dr Pamela Russell (Prince of Wales Hospital, AU).

The LDLp-588luc plasmid is a luciferase reporter plasmid containing the *LDLR* promoter region [19], and was a gift from Dr Thierry Grand-Perret (GlaxoSmithKline, FR). The LDLp-mutSRE plasmid was derived from the LDLp-588luc plasmid using site-directed mutagenesis, as described below. The phRL-TK plasmid expresses *Renilla* luciferase constitutively, serving as a transfection control, and was obtained from Promega (Annandale, NSW, AU).

The IgG-1C6 mouse anti-SREBP-2 primary antibody [20] was obtained from BD Biosciences (North Ryde, NSW, AU). The B-5-1-2 mouse anti-α-tubulin primary antibody [45] was obtained from Sigma-Aldrich (Castle Hill, NSW, AU). Peroxidase-conjugated AffiniPure donkey anti-mouse secondary antibody was obtained from Jackson ImmunoResearch Laboratories (Sydney Markets, NSW, AU).

DMSO, NaCl, and all solvents (analytical grade) used for thin layer chromatography were obtained from Ajax FineChem (Taren Point, NSW, AU). Glycerol was obtained from B.D.H. Chemicals (Port Fairy, Vic, AU). 40% Acrylamide/bis-acrylamide solution, and Precision Plus Protein Kaleidoscope standard biomarkers were obtained from Bio-Rad Laboratories (Regents Park, NSW, AU). Isopropanol, methanol, HCl, and NaOH were obtained from Crown Scientific (Moorebank, NSW, AU). RPMI medium 1640, fetal calf serum (FCS), and penicillin-streptomycin were obtained from Invitrogen (Mt Waverley, Vic, AU). 1-bromo-3-chloropropane, bromophenol blue, bovine serum albumin, compactin (mevastatin), β-mercaptoethanol, l,l′-dioctadecyl-3,3,3′,3′-tetramethylindocarbocyanine perchlorate (DiI), Dulbecco's PBS, primers, SDS, Tween20, and Tris-base were obtained from Sigma-Aldrich (Castle Hill, NSW, AU). 25-HC was obtained from Steraloids (RI, USA). [1-^14^C]-acetic acid (specific activity: 56.0 mCi/mmol) was obtained from GE Healthcare (Rydalmere, NSW, AU).

FCLPDS was prepared from FCS as described previously [22] and diluted to 30 mg/ml with 0.15 M NaCl. LDL was prepared by standard ultracentrifugation techniques (d = 1.019−1.063 g/ml) from the plasma of healthy male volunteers [46]. PBST was 0.1% (v/v) Tween20 in PBS. DiI-labeled LDL (DiI-LDL) was prepared by incubating DiI with undesalted LDL (3∶10 w/w ratio) for 18 h at 37°C, and purifying the resulting DiI-LDL using the PD-10 chromatography column (GE Healthcare, Rydalmere, NSW, AU), according to the manufacturer's instructions.

### Cell culture

PC-3, LNCaP, and FB cells were maintained in Medium A (RPMI 1640, supplemented with 10% [v/v] FCS, 100 U/mL penicillin and 100 µg/mL streptomycin [PS]). Transfections were performed in Medium B (Medium A without antibiotics) and treatment was in Medium C (RPMI 1640, supplemented with 10% [v/v] FCLPDS and PS). Prior to plating PC-3 or LNCaP cells, plates and dishes were treated with 0.1 µg/ml poly-L-lysine (R&D Systems, Gymea, NSW, AU) for 1 h to enhance cellular adhesion. PrEC cells were maintained and treated in PrEGM (Lonza, Mt Waverley, Vic, AU), and transfected in PrEGM without antibiotics. Similar results were obtained when LNCaP cells were treated in PrEGM or Medium C (data not shown). Cells were plated to obtain 70–90% confluence at the end of the treatment.

### Quantitative real-time reverse-transcription polymerase chain reaction (qRT-PCR)

Following treatment in experiments examining the expression of SREBP-2 target genes, total RNA was harvested, reverse transcribed to cDNA, and mRNA levels determined (from cDNA) by qRT-PCR, as previously described [47]. Primers used to amplify human *LDLR*, *HMGCR*, and *PBGD* cDNA have been previously described [46], [48]. Amplification data was analyzed using Rotor-Gene Version 6.0 (Build 27) (Corbett Research, Mortlake, NSW, AU). Melting curve analysis was performed to confirm the production of a single product in each reaction. The mRNA expression levels of *HMGCR* and *LDLR* genes were normalized to *PBGD* and made relative to the vehicle condition using the ΔΔC_t_ method.

### Site-directed mutagenesis

In order to examine activity more specific to SREBP-2, the SRE within the *LDLR* promoter region of the LDLp-588luc reporter plasmid [19] was mutated using site-directed mutagenesis. The SRE sequence was mutated from 5′-ATCACCCCAC-3′ to 5′-ATCACGGCTC-3′ (mutations underlined), previously shown to prevent SREBP-2 binding [49]. This was performed using 50 ng template DNA and the iProof High-Fidelity DNA polymerase (Bio-Rad, Regents Park, NSW, AU), according to the manufacturer's instructions, with the addition of 6% (v/v) DMSO to enhance reaction efficiency. The forward primer was 5′-AAGACATTTGAAAATCACGGCTCTGCAAACTCCTCCCCCTG-3′ (mutations underlined), and the reverse primer was the forward primer's reverse-complement. The reaction product was then treated using the QuikChange II XL Site-Directed Mutagenesis Kit (Stratagene, CA, US), according to the manufacturer's instructions. The mutation was confirmed by sequencing. The mutant plasmid was labeled ‘LDLp-mutSRE’.

### Luciferase assay

For each experiment, cells were plated in two 100 mm dishes and transfected with either LDLp-588luc or LDLp-mutSRE (10 µg), and both cotransfected with phRL-TK (1 µg) as a transfection control. These transfections were performed using Lipofectamine LTX transfection reagent, Plus reagent, and OptiMEM I (all obtained from Invitrogen, Mt Waverley, Vic, AU), according to the manufacturer's instructions. After 4 h, cells were then split into 24-well plates in Medium C (LNCaP, PC-3, FB) or PrEGM (PrEC), and allowed to adhere overnight. Since LNCaP cells adhere poorly to culture dishes [30], the washing and media-refreshment were avoided for all cell-lines. Rather, the treatment was delivered in a small quantity of plating medium, added to the existing media in the wells.

After treatment, the luciferase assays were conducted using the Dual-Luciferase Reporter Assay System (Promega, Annandale, NSW, AU), according to the manufacturer's instructions. Luciferase activity was measured using a Veritas luminometer (Turner Designs, CA, US), and expressed as change in firefly luciferase activity relative to *Renilla* luciferase activity. The mutant promoter (LDLp-mutSRE) luciferase activity was subtracted from the wild-type promoter (LDLp-588luc) luciferase activity to obtain SRE-specific activity for each experimental condition.

### Western blotting

For experiments where SREBP-2 processing was examined, cells were harvested for protein and protein aliquots (30 µg) were subjected to 10% (w/v) SDS-PAGE, and transferred to Trans-Blot transfer medium (Bio-Rad, Regents Park, NSW, AU), as previously described [47]. Membranes were blocked in 5% (w/v) skim milk, 5% (v/v) FCS PBST, for 1 h at room temperature. This was followed by incubation in primary antibody for 2 h (for anti-SREBP-2) or 1 h (for anti-α-tubulin) at room temperature, washing with PBST 3 times for 10 min, incubation in secondary antibody for 1 h at room temperature, and washing with PBST 3 times for 10 min. Antibodies were visualized on Hyperfilm (GE Healthcare, Rydalmere, NSW, AU) using the ECL detection system (Millipore, North Ryde, NSW, AU). The anti-SREBP-2 antibody (IgG-1C6) detects the C-terminal product of mature SREBP-2 at ∼68 kDa [20], confirmed by inputting the amino acid sequence of the human SREBP-2 C-terminal fragment (SREBP-2 sequence obtained from UniProt Accession Number Q12772) into Compute pI/Mw Tool (ExPASy, Geneva, CH). Between antibodies, membranes were treated with a stripping buffer (25 mM glycine [pH 2], 1.5% [w/v] SDS). Films were scanned using HP Scanjet G3010 and accompanying software (Hewlett-Packard, CA, USA).

### LDL uptake assay

To quantify LDLR activity, the LDL uptake assay was performed as previously described [50], [51], with modifications. Briefly, cells were seeded in duplicate plates in Medium C (FB, PC-3, LNCaP) or PrEGM (PrEC) and allowed to adhere overnight. Treatment was delivered in a small quantity of plating medium, added to the existing media in the wells – there were triplicate wells for each treatment condition, performed in duplicate. After treatment, DiI-LDL was added to each well (obtaining a final DiI concentration of 10 µg/ml protein), and for 2 h, one set of cells was incubated at 37°C and the other at 4°C. Cells were then washed once with PBS and lysed with lysis buffer (0.1 M NaOH, 0.1% [w/v] SDS). Cell lysate was assayed for fluorescence using an Fmax microplate spectrofluorometer (Molecular Devices, CA, US) at excitation λ = 544 nm and emission λ = 612 nm, and for protein content using the BCA assay kit (Pierce, Paddington, Qld, AU). Fluorescence was normalized to protein content for each sample. For each treatment condition, the difference between the normalized DiI-LDL fluorescence of cells incubated at 37°C and 4°C determined internalized DiI-LDL.

### Cholesterol synthesis assay

Following treatment, cells were labeled with 1 µCi/well [1-^14^C]-acetic acid for 2 h in the presence of treatment, after which lipids were extracted and separated by thin layer chromatography, as previously described [46], with the exception that the mobile phase used in the chromatography was hexane:diethyl ether:glacial acetic acid (60∶40∶1, v/v/v). After development, the band corresponding to cholesterol was visualized using the FLA-5100 phosphorimager (Fujifilm, Tokyo, JP). The relative intensities of bands were quantified using Sciencelab ImageGauge 4.0 software (Fujifilm, Tokyo, JP).

### Statistical analysis

Data are presented as means, with errors bars as SEMs. Two-sample *t*-tests were used to assess statistical differences between treatments: *p<*0.05 (two-tailed) was considered significant. Statistical tests were performed using Microsoft Excel 2003 (Microsoft, USA).

## Supporting Information

Figure S1 The wild-type luciferase construct (LDLp-588luc) responds to changing sterol levels, whilst the mutant luciferase construct (LDLp-mutSRE) does not. Cells were transfected as described in Materials and Methods. Treatment included the statin compactin (CPN, 5 µM), oxysterol 25-HC (1 µg/ml), or LDL (50 µg/ml) for 24 h. The luciferase assay was performed as described in Materials and Methods. The firefly:*Renilla* ratio of each treatment was normalized to vehicle condition to obtain relative luciferase activity. Data are mean +SEM, from 3 separate experiments for each cell-line. Each experiment was performed with triplicate wells per condition.
(0.94 MB TIF)

Figure S2 Sterol-mediated regulation of SREBP-2 target genes exists in PC-3 cells over varying time periods. PC-3 cells were treated with the statin compactin (CPN, 5 µM) or oxysterol 25-HC (1 µg/ml) for the times indicated. The mRNA expression of the *LDLR* and *HMGCR* genes were analyzed as described in Fig. 1, and made relative to the 0 h vehicle condition. Data are mean + SEM, from 3 separate experiments. Each experiment was performed with triplicate wells per condition. * *p*<0.05, ** *p*<0.01, two-sample *t*-test versus vehicle condition at that respective time-point.
(0.38 MB TIF)
