# Supplementary figures and images for: Cholesterol Homeostasis in Two Commonly Used Human Prostate Cancer Cell-Lines, LNCaP and PC-3
Source: PLoS One. 2009 Dec 30;4(12):e8496. doi: 10.1371/journal.pone.0008496 (PMC2794383; doi:10.1371/journal.pone.0008496)

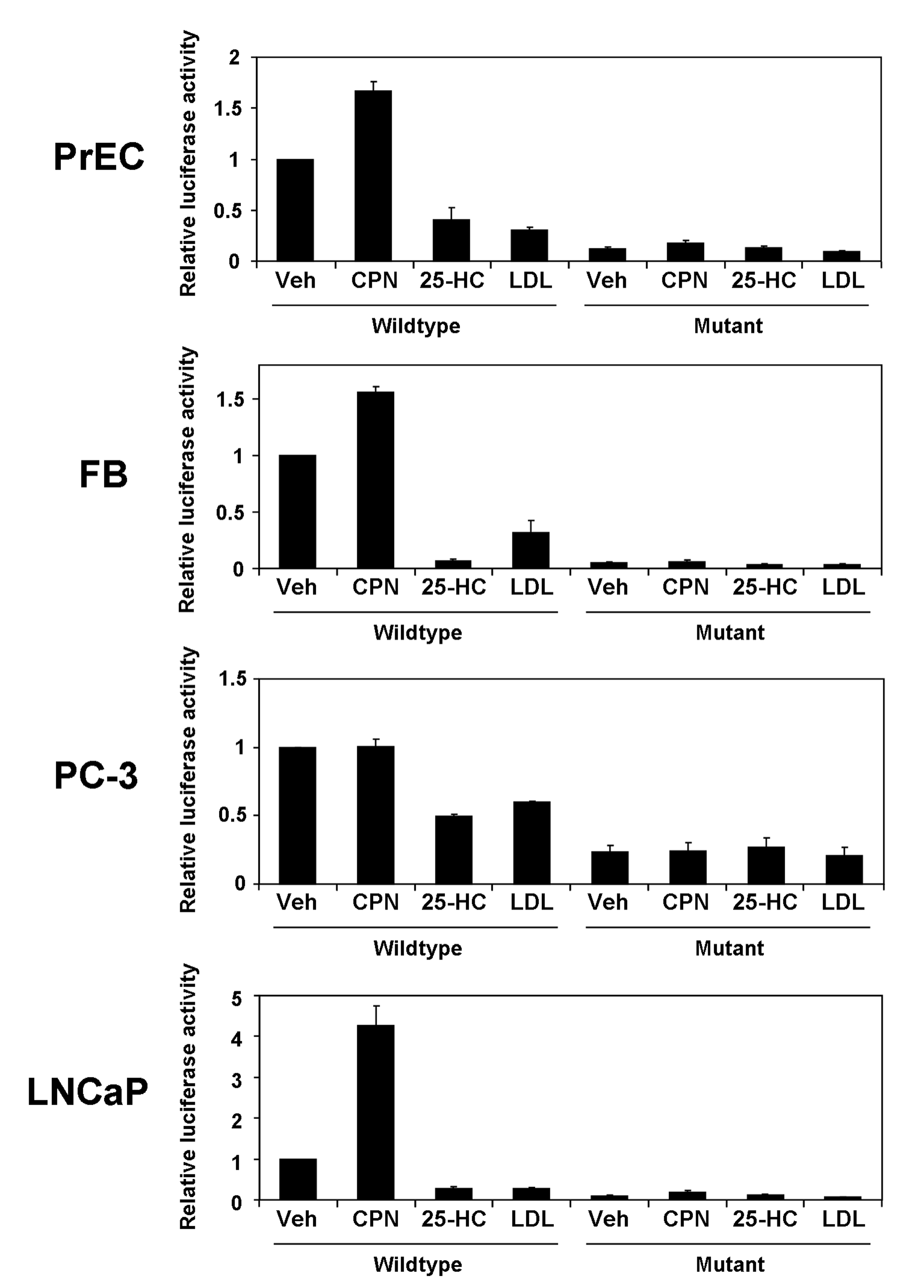

Supplement: Figure S1 — The wild-type luciferase construct (LDLp-588luc) responds to changing sterol levels, whilst the mutant luciferase construct (LDLp-mutSRE) does not. Cells were transfected as described in Materials and Methods. Treatment included the statin compactin (CPN, 5 µM), oxysterol 25-HC (1 µg/ml), or LDL (50 µg/ml) for 24 h. The luciferase assay was performed as described in Materials and Methods. The firefly:Renilla ratio of each treatment was normalized to vehicle condition to obtain relative luciferase activity. Data are mean +SEM, from 3 separate experiments for each cell-line. Each experiment was performed with triplicate wells per condition. (0.94 MB TIF) [file pone.0008496.s001.tif]

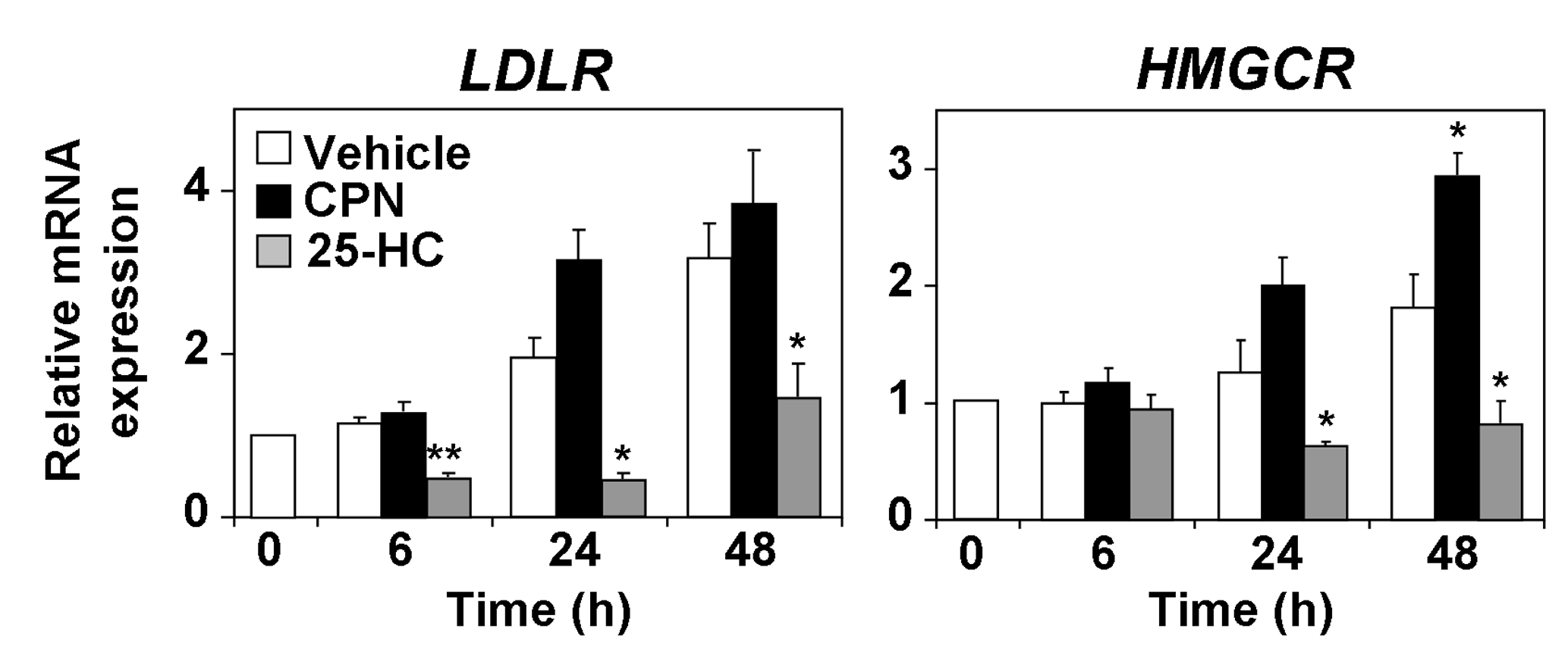

Supplement: Figure S2 — Sterol-mediated regulation of SREBP-2 target genes exists in PC-3 cells over varying time periods. PC-3 cells were treated with the statin compactin (CPN, 5 µM) or oxysterol 25-HC (1 µg/ml) for the times indicated. The mRNA expression of the LDLR and HMGCR genes were analyzed as described in Fig. 1, and made relative to the 0 h vehicle condition. Data are mean + SEM, from 3 separate experiments. Each experiment was performed with triplicate wells per condition. * p<0.05, ** p<0.01, two-sample t-test versus vehicle condition at that respective time-point. (0.38 MB TIF) [file pone.0008496.s002.tif]
